# Removal of high concentrations of zinc, cadmium, and nickel heavy metals by *Bacillus* and *Comamonas* through microbially induced carbonate precipitation

**DOI:** 10.1007/s10532-025-10131-7

**Published:** 2025-05-05

**Authors:** Adharsh Rajasekar, Cailin Zhao, Suowei Wu, Raphinos Tackmore Murava, Eyram Norgbey, Armstrong Ighodalo Omoregie, Charles K. S. Moy

**Affiliations:** 1 Jiangsu Key Laboratory of Atmospheric Environment Monitoring and Pollution Control (AEMPC), Collaborative Innovation Center of Atmospheric Environment and Equipment Technology (CIC-AEET), Nanjing University of Information Science &Technology, Nanjing, 210044 China; 2 School of Geography and Environmental Science, University of Reading, Reading, RG67BE UK; 3 Centre for Borneo Regionalism and Conservation, University of Technology Sarawak, No. 1 Jalan University, 96000 Sibu, Sarawak Malaysia; 4 Department of Civil Engineering, Xi’an Jiaotong-Liverpool University, Suzhou, 215123 Jiangsu P. R. China

**Keywords:** Heavy metals, MICP, Heavy metal carbonates, Applied microbiology, Ureolytic bacteria

## Abstract

Heavy metal pollution in urban freshwater, driven by anthropogenic activities, poses significant risks to aquatic ecosystems and human health due to its toxicity and persistence. Recently, urease-producing bacteria have gained attention for their ability to remove heavy metals through microbial-induced carbonate precipitation (MICP). In this study, eight urease-producing bacteria were exposed to individual solutions of zinc (Zn^2+^), cadmium (Cd^2+^), and nickel (Ni^2+^) at concentrations ranging from 0 to 6 mM to assess their resistance. Three strains—*Bacillus subtilis* HMZC1 (B2), *Bacillus sp.* HMZCSW (B6), and *Comamonas sp.* HMZC (B11)—survived at 4 mM and 6 mM, while most others could not tolerate 4 mM. Their urea-degrading ability was tested at different pH levels, identifying an optimal pH of 7 for MICP. Heavy metal carbonate precipitation experiments at 4 mM and 6 mM revealed that all three strains achieved > 93% removal of Zn^2+^, Ni^2+^, and Cd^2+^ within 72 h. *Comamonas sp.* HMZC exhibited the highest efficiency, achieving > 95% removal of certain heavy metals at 6 mM. Statistical analysis using one-way ANOVA revealed significant differences (p < 0.05) in heavy metal removal efficiencies among the strains for certain treatment conditions (Cd^2+^ and Zn^2+^ at 4 mM), although not all comparisons reached statistical significance. Scanning Electron Microscopy and X-ray Diffraction confirmed the morphology and composition of the precipitated heavy metal carbonates. Our findings demonstrate that urease-producing bacteria can effectively immobilize multiple heavy metals, highlighting the MICP process as a practical and sustainable biological approach for ecological restoration and wastewater treatment.

**Supplementary Information:**

The online version contains supplementary material available at 10.1007/s10532-025-10131-7.

## Introduction

The rise in industrial standards and large-scale resource use has driven socio-economic development and disrupted ecosystems on an unprecedented scale. Potentially toxic elements (PTEs) like cadmium (Cd), zinc (Zn), and nickel (Ni) are now prevalent in freshwater ecosystems worldwide (Halawani et al. 2022; Qin and Tao 2022). These heavy metals pose significant risks as they accumulate in plants and animals through bioaccumulation and biomagnification, leading to acute or chronic health issues in humans, including cancer, neurological diseases, and metabolic damage (Vareda et al. 2019). Assessing PTE risks is challenging due to complex exposure routes, interactions, and poorly understood biogeochemical processes that govern their mobility across water, soil, air, and biological systems (Lestiani et al. 2023). Reducing heavy metal mobility and bioavailability is a key goal of environmental remediation (Elnabi et al. 2023). Microbial remediation, which leverages microorganisms’ metabolic processes to adsorb, mineralize, or transform heavy metals, offers a cost-effective and eco-friendly solution (Kumar et al. 2021). Among these methods, microbially induced carbonate precipitation (MICP) stands out as a sustainable alternative to conventional techniques, minimizing chemical waste and energy consumption (Rajasekar et al. 2025). By capturing heavy metals in stable carbonate forms, MICP mitigates contamination and supports ecosystem restoration, making it a promising tool for addressing the growing threat of PTE pollution.

Microbially induced calcium/carbonate precipitation (MICP) is a natural biomineralization process driven by microbial metabolic activities. Depending on the metabolic pathways, MICP can occur through photosynthesis, methane oxidation, sulfate reduction, amino acid ammonification, urea degradation, or nitrate reduction. Among these, carbonate precipitation by ureolytic bacteria has gained significant attention (Mondal and Ghosh 2019; Seifan and Berenjian 2019). Ureolytic bacteria produce urease, an enzyme that catalyzes the breakdown of urea into ammonia and carbonic acid. This reaction increases the pH and shifts the carbonate equilibrium, forming carbonate ions (Anbu et al. 2016; Krajewska, 2018). Negatively charged bacterial cell membranes or extracellular polymeric substances (EPS) trap calcium or heavy metal ions, providing nucleation sites for carbonate mineral precipitation. The morphology of the resulting crystals depends on factors like bacterial concentration, temperature, and pH (AJ et al. 2013; Castro-Alonso et al. 2019). Heavy metal ions with atomic structures similar to calcium can substitute into the calcite lattice, forming co-precipitates (Zhang et al. 2023a). Alternatively, carbonate ions may directly bind with heavy metals to form insoluble minerals (Torres-Aravena et al. 2018; Kumar et al. 2023). This process also sequesters CO₂, making MICP an eco-friendly technique for soil and water remediation, metal recovery, and wastewater treatment (Omoregie et al. 2021; Song et al. 2022a). Dong et al. (2023) demonstrated the role of Ca^2^⁺ concentration in enhancing heavy metal removal via co-precipitation using *Sporosarcina pasteurii*. Overall, MICP offers a sustainable solution for environmental remediation by immobilizing contaminants and reducing their bioavailability.

1 $$CO\left( {NH_{2} } \right)_{2} + H_{2} O\xrightarrow{{Urease}}NH_{2} COOH + NH_{3}$$

2 $$\begin{array}{*{20}c} {NH_{2} COOH + H_{2} O \to NH_{3} + H_{2} CO_{3} } \\ \end{array}$$

3 $$\begin{array}{*{20}c} {CO\left( {NH_{2} } \right)_{2} + 2H_{2} O \to 2NH_{3} + H_{2} CO_{3} } \\ \end{array}$$

4 $$\begin{array}{*{20}c} {2NH_{3} + 2H_{2} O \leftrightarrow 2NH_{4}^{ - } + 2OH^{ - } } \\ \end{array}$$

5 $$\begin{array}{*{20}c} {H_{2} CO_{3} \leftrightarrow HCO_{3}^{ - } + H^{ + } \leftrightarrow CO_{3}^{2 - } + 2H^{ + } } \\ \end{array}$$

6 $$\begin{array}{*{20}c} {Ca \left( {metal ions} \right)^{2 + } + Cell + CO_{3}^{2 - } \to Cell - Ca \left( {metal ions} \right)CO_{3} \downarrow } \\ \end{array}$$

Many previous studies have focused on isolating efficient ureolytic microorganisms for heavy metal removal. For instance, Tang et al. (2023) isolated *Penicillium chrysogenum* CS1, a detoxifying fungal strain capable of removing Pb^2^⁺ and Cr^2^⁺ from both solution and soil matrices. While ureolytic fungi have been explored, most research centers on bacteria, with *Sporosarcina pasteurii* being one of the most studied ureolytic species (Table S1). Investigations into ureolytic bacteria typically examine their resistance to heavy metal toxicity, changes in urease activity, and pH dynamics during microbial-induced carbonate precipitation (MICP). Additionally, researchers have explored how initial heavy metal concentrations and exposure durations influence removal rates (Zeng et al. 2022; Yina et al. 2021).

Temperature and starting pH are critical factors influencing bacterial growth and urease activity. Previous studies suggest an optimal temperature range of 20–37 °C and a starting pH of 7–8 for MICP (Anbu et al. 2016; Rajasekar et al. 2021). However, preferences vary among species, with most favoring slightly acidic to neutral conditions (pH = 7). Despite these insights, as shown in Table S2, most studies report heavy metal removal efficiencies of 75–100% under controlled conditions, but these results are typically achieved at low concentrations (< 2 mM). This highlights a significant gap in addressing high-concentration environments. Recent efforts have begun focusing on isolating ureolytic bacteria capable of tolerating high heavy metal concentrations. For example, Li et al. (2021) identified *Lysinibacillus* sp. strains tolerant to 100 ppm Cu and 1,000 ppm Pb from an e-waste site. Similarly, Qiao et al. (2021) isolated four urease-producing strains from a mine in Sichuan, China, evaluating their tolerance and removal efficiency for Cu, Zn, Ni, and Cd. However, most MICP studies focus on single-metal systems, leaving multi-metal scenarios underexplored. To advance MICP applications, isolating bacterial strains with high tolerance to heavy metal toxicity and the ability to remove multiple metals simultaneously is crucial. Such efforts will enhance MICP as a sustainable solution for remediating environments contaminated with high levels of heavy metals.

**Fig. 1:**
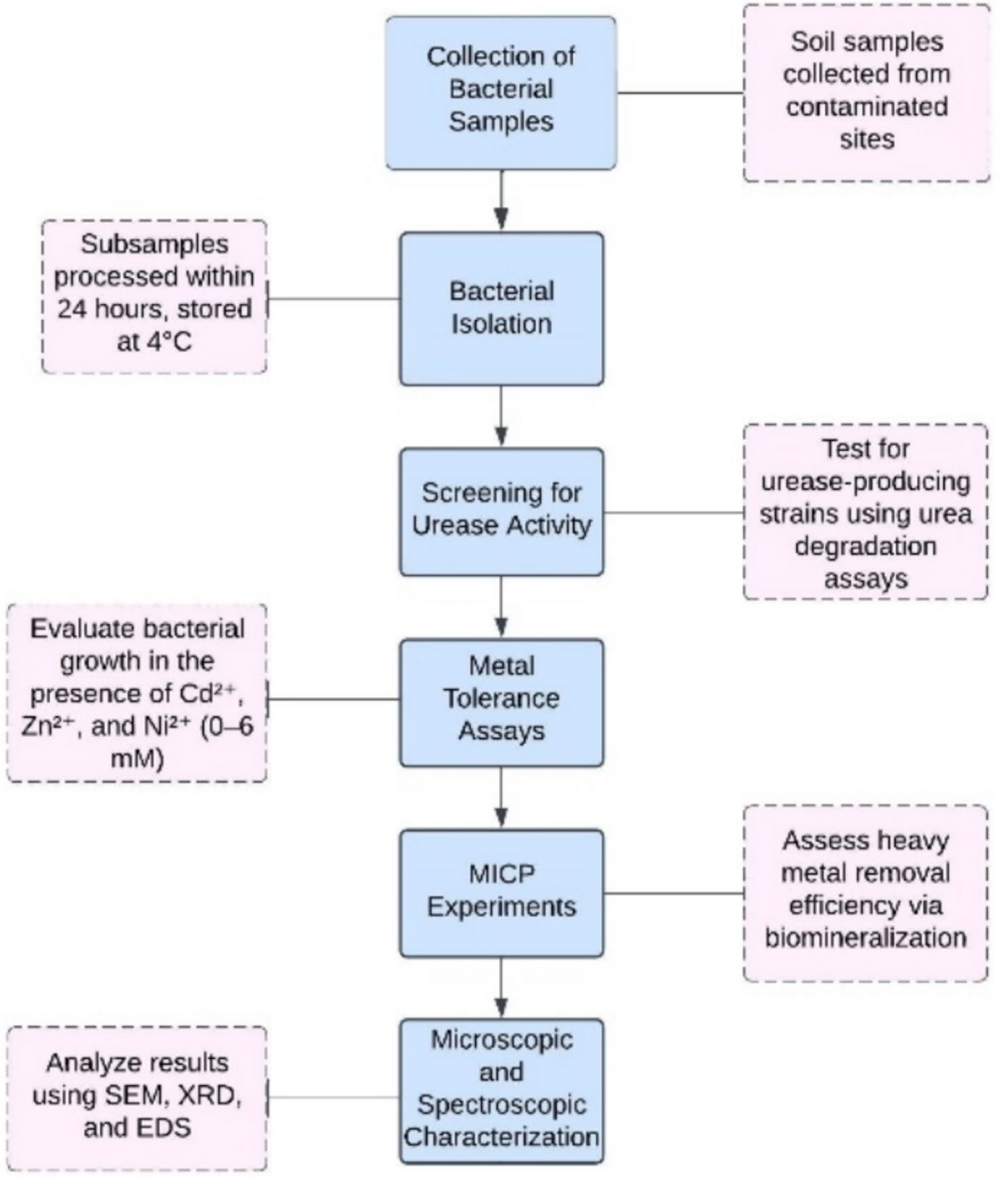
Flowchart of the experimental approach

The objectives of this study are 1) to isolate efficient ureolytic bacteria with strong metal toxicity tolerance in Qunying River, Nanjing University of Information Science and Technology, China, 2) to analyze the urea decomposition changes during the 96-h growth of different selected bacteria and determine the best initial pH for MICP, 3) to assess the heavy metal removal ability of selected strains under high concentrations of the three focused heavy metals (Cd, Zn, and Ni). Bacteria with high viability and remediation potential are crucial for remediating contaminated environments. The experimental approach taken in this study is depicted in Fig. 1, which provides a detailed flowchart outlining the sequence of experiments and the methodology used to achieve the research objectives. This study aims to investigate the two-way interaction between environments contaminated with high PTEs and ureolytic bacteria with potent heavy metal toxicity tolerance ability and to provide a feasible solution for the future application of MICP technology on remediation projects in severe heavy metal contaminated environments.

## Materials and methods

### Screening and identification of urease-producing bacterial strains

A total of 28 indigenous bacteria were isolated from a polluted catchment (Qunying River) near Pukou, Nanjing, China. The samples of surface water (0–5 cm) were collected in High-density polyethylene bottles. The samples were transported to the lab at 4 °C for bacterial isolation. These bacteria were initially isolated using the serial dilution and pour plate method. The pour plate method was performed using Nutrient agar (NA) (10 g/L peptone, 3 g/L beef extract, 5 g/L NaCl, and 15 g/L agar) (hopebio™, China). Upon their isolation, pure bacteria cultures were inoculated in Christensen- Urea agar (Oualha et al. 2020; Leeprasert et al. 2022). The agar was autoclaved at 121 °C for 15 min. The urea solution was added to the agar when the temperature was close to 45 ± 3 °C. The bacteria from the group were chosen based on their ability to change the color of agar from yellow-orange to pink-red, as this indicates their ability to break down urea effectively. The incubation was performed at 30 °C for 96 h. The control had no bacteria in it.

### Secondary screening of ureolytic bacteria based on urea degradation

Eight isolated bacteria were tested for their ability to degrade urea by inoculating them into a medium (100 mL) containing 20 g/L urea (SCPTM, China) and nutrient broth. The nutrient broth (NB) was autoclaved at 121 °C for 15 min. Urea was added to the NB using a 50 mL syringe connected to a 0.22 µm filter to avoid contamination. Nutrient Broth-Urea (NBU) solution is a combination of NB and urea. The OD_600_ of the bacteria suspension was 1.0. OD_600_ refers to optical density at 600 nm, a measure of bacterial growth. This experiment was conducted at various pH levels, namely 6, 7, and 8, to test their ability for growth and urea degradation and also for future field applications. Different pH levels were tested to determine the optimal conditions for urea degradation and bacterial growth, which are crucial for MICP. The experiment was conducted at 140 rpm at 30 °C. The urea degradation was determined using the para-dimethyl amino benzaldehyde (PDAB) (98% purity) method (Knorst et al. 1997). As mentioned by the protocol, 1 mL of PDAB was added to 4 mL of diluted urea-containing solution. A standard curve was constructed using multiple concentrations of urea. The absorbance of the solution was measured at 422 nm using a UV–Vis spectrophotometer (Thermo Scientific Genesys 10S, Waltham, MA, USA). The samples were taken every 24 h until urea could not be detected in one of the pH levels. All experiments were conducted in triplicate.

### Metal toxicity test

Eight bacteria were subjected to multiple Zinc chloride (ZnCl_2_) (99.9% trace metals basis, Rhawn™, China), Cadmium chloride (CdCl_2_) (99.99% trace metals basis, Aladdin™, China), and Nickel chloride hexahydrate (NiCl_2_.6H_2_O) (99.9% trace metals basis, Aladdin™, China) concentrations. The concentrations used were 1, 2, 4, and 6 mM. A heavy metal solution was incorporated into the nutrient agar in a Petri plate. After the agar solidified, 10 µL of a bacterial suspension (OD_600_ = 1.0) was added to the plate, which was then incubated at 30 °C for seven days to monitor bacterial growth (Kumari et al. 2016; Bai et al. 2021b; Disi et al. 2022). The control had no bacteria in it. The ureolytic strains were selected based on their demonstrated resistance to high concentrations of heavy metals, as indicated by their minimum inhibitory concentration (MIC), which refers to the lowest concentration of a heavy metal that completely inhibits bacterial growth. This resistance is critical for their application in bioremediation.

### Genomic identification of the heavy metal-resistant bacteria

The dominant strain with heavy metal resistance was selected for genomic identification. The genomic DNA of the strains was isolated from the pure cultures using a DNA extraction kit. The gene was amplified using the primers 27F (5′-AGAGTTTGATCMTGGCTCAG-3′) and 1492R(5′-GGTTACCTTGTTACGACTT-3′) (Muyzer et al. 1993; Frank et al. 2008). Polymerase Chain reaction (PCR) was performed on the template DNA by following the protocol mentioned by (Rajasekar et al. 2018). The PCR product was assessed in 0.8% agarose gel electrophoresis. The PCR products were recovered using the Springen Agarose Gel Magnetic Bead Method DNA Recovery Kit (MD003-100). Purified products were sequenced in both directions by an ABI3730-XL sequencer (Thermo Fisher Scientific, Waltham, MA, USA). Nucleotide sequences were edited using DNAstar Lasergene (version 7.1) software. Basic Local Search Alignment Tool (BLAST) analysis was performed to compare the sequences with available DNA sequences from the National Center for Biotechnology Information (NCBI) database (Altschul et al. 1990). The accession numbers for the three bacterial strains are B2 (OQ826692) (*Bacillus subtilis* HMZC1, 86.48%, 1229 basepairs), B6 (OQ826707) (*Bacillus sp.* HMZCSW, 98.32%, 1198 basepairs), and B11 (OQ826684) (*Comamonas sp.* HMZC, 99.93%, 1417 basepairs). A phylogenetic tree was constructed using the neighbor-joining method (Figure S1).

### Heavy metal carbonate studies

Three efficient heavy metal-resistant bacteria were chosen for heavy metal carbonate studies based on the urea degradation result. The bacterial suspension was added to a 100 ml solution containing 4 mM ZnCl_2,_ autoclaved nutrient broth, and filter-sterilized urea (20 g/L). The same procedure was repeated to 4 mM CdCl_2_, NiCl_2_.6H_2_O, and 6 mM ZnCl_2_, CdCl_2_, and NiCl_2_.6H_2_O. The OD of the bacteria suspension was 1.0. The experimental conditions include pH 7, temperature 30 °C, and mixing at 180 rpm. The experiment was conducted for a total of 96 h. The 96-h timeframe was chosen as it corresponds to the standard duration used in our urea degradation test. While urease activity or urea degradation was not directly measured during the heavy metal tolerance tests, this timeframe was selected to ensure sufficient bacterial growth, urea degradation, and potential carbonate precipitation under experimental conditions. The control had no bacteria in it. All experiments were conducted in triplicate. After the experiment, the solution was filtered through a 0.45 µm filter to collect the precipitates and dried at 48 °C for 48 h for quantification and qualification analysis. Atomic Absorption Spectroscopy was used to quantify the leftover heavy metal in the solution using a Flame Atomic Absorption Spectrometer (ZEEnit 700P, Analytik Jena GmbH + Co. KG, Jena, Germany). Before the quantification, the solution was appropriately digested with 2% nitric acid to stabilize heavy metals and prevent adsorption or precipitation (Cheng et al. 2024). A series of calibration standards were prepared from stock solutions. The heavy metal removal rates of the selected strains were calculated from the difference between the initial and final elemental concentrations in the solution (Eq. (7)).

7 $${\text{Removal capacity }}\left( \% \right) \, = \, \left( {{\text{C}}_{0} - {\text{C}}_{{{96}}} } \right)/{\text{C}}_{0} \times {1}00$$

C_0_ and C_96_ represent the initial and final (96 h) concentrations of heavy metals (mM), respectively.

### Characterization analysis

Scanning Electron Microscope (SEM) (thermo-field emission FEI Quanta 400FEG (Thermo Fisher Scientific, Waltham, MA, USA)) was used to visualize the morphology of the heavy metal carbonates precipitated during the experiment. X-ray Diffractometer (XRD) (SmartLab (9) diffractometer (Rigaku, Japan) with an ultra-high speed detector (40 kV and 150 mA) scanning from 5° to 90° at a step rate of 8°/min was performed to characterize the heavy metal carbonates. The XRD peaks were compared with reference data from the International Centre for Diffraction Data (ICDD) database to identify the crystalline phases of the precipitates (Kabekkodu et al. 2024).

### Statistical analysis

The results are shown as means ± standard deviation. All data were analyzed using one-way ANOVA followed by Tukey’s posthoc test to compare the effects of different bacterial strains on removal efficiency and urea degradation using Origin 2024b. We confirmed that the data met the assumptions of normality (Shapiro–Wilk test) and homoscedasticity (Levene’s test) before performing ANOVA. The confidence interval was 95%, and the error bars in the figures represent the standard deviations from the mean performed using Microsoft Excel 2019.

## Results and discussion

### Heavy metal toxicity tolerance

The problem of anthropogenic heavy metal pollution of urban rivers and lakes is becoming increasingly serious, and heavy metals in water bodies affect the population structure of microorganisms to some extent. Although the urease-induced MICP process can reduce the concentration of heavy metals in the environment by producing heavy metal precipitates, no studies have been conducted to confirm whether urea hydrolysis is one of the mechanisms of tolerance of PTEs. We do not believe that the MICP process is the primary mechanism by which microorganisms resist heavy metals, as the urease in the MICP process may be an extracellular enzyme, or it could be potentially released during the rupture of the cell membrane after the microorganism has died. Since the heavy metals are adsorbed to the surface of the bacterial cell wall, they also serve as a nucleation site for carbonate binding, which results in the dormancy or death of the microorganism (Sheng et al. 2022). It is not ideal for determining the MICP ability of a strain by its heavy metal tolerance alone. However, it is undeniable that the heavy metal resistance of a strain determines its potential to survive in different PTEs contaminated environments and the scope of its application in environmental remediation (Henao and Ghneim-Herrera 2021).

Previous studies have confirmed that Cd is the most biotoxic among Cd, Zn, and Ni, followed by Zn and Ni (Carpio et al. 2018; Qiao et al. 2021). Therefore, the MIC of the selected strains was used to determine the heavy metal resistance of the selected strains. The MIC results for Cd, Ni, and Zn showed (Table 1) that most strains were not observed to grow at 6 mM (MIC = 6 mM). There are two main ways to test the microorganism’s tolerance to heavy metals: one is to quantify the effect of heavy metals on bacterial growth by measuring the OD_600_ of NB media containing heavy metals, and the other is to measure the inhibition of colony diameter or growth by heavy metals through NA media (Bai et al. 2021b; He et al. 2019). Taking the concentration of heavy metals that completely inhibits bacterial growth as the MIC, the results vary using different methods or different times of incubation. Several studies have tested heavy metal resistance in NA media and obtained high MIC values. Most bacteria studied for heavy metal carbonate precipitation could not grow above 2 mM for Cd, Ni, and Zn (Bai et al. 2021b; Kumar et al. 2023; Qiao et al. 2021). However, Jalilvand et al. (2019) reported ureolytic strains isolated from Iranian mining soils that were tolerant to Cd concentrations of up to 11 mM. In our results, the three most tolerant strains, B2, B6, and B11, were tolerant up to 6 mM (Table 1). A review conducted by Tamayo-Figueroa et al. (2019) indicated that Cd, Ni, and Zn are the most potent heavy metals to inhibit bacterial growth, even as low as 1 mM. The B2, B6, and B11 strains have shown much more resistance toward higher heavy metal concentrations. These bacteria will be further studied for urea degradation and heavy metal carbonate precipitation.

**Table 1:** Toxicity Tolerance of Urease-Positive Bacterial Strains to Heavy Metals (Cd^2^⁺, Ni^2^⁺, and Zn^2^⁺): Growth Responses at Different Concentrations (0–6 mM). Growth was observed ( +) or completely inhibited (-)

Strain	Cd^2+^ (mM)					Ni^2+^ (mM)					Zn^2+^ (mM)				
	0	1	2	4	6	0	1	2	4	6	0	1	2	4	6
Control	−	−	−	−	−	−	−	−	−	−	−	−	−	−	−
B2 (*Bacillus subtilis* HMZC1)	+	+	+	+	+	+	+	+	+	+	+	+	+	+	+
B3	+	+	+	+	−	+	+	−	−	−	+	+	+	−	−
B6 (*Bacillus sp.* HMZCSW)	+	+	+	+	+	+	+	+	+	+	+	+	+	+	+
B9	+	+	+	−	−	+	+	+	+	−	+	+	+	−	−
B10	+	+	−	−	−	+	+	+	−	−	+	+	+	−	−
B11 (*Comamonas sp.* HMZC)	+	+	+	+	+	+	+	+	+	+	+	+	+	+	+
B12	+	+	−	−	−	+	+	+	−	−	+	+	+	−	−
B16	+	+	+	−	−	+	+	+	−	−	+	+	+	−	−

### Urea degradation capacity of different selected bacteria

**Fig. 2:**
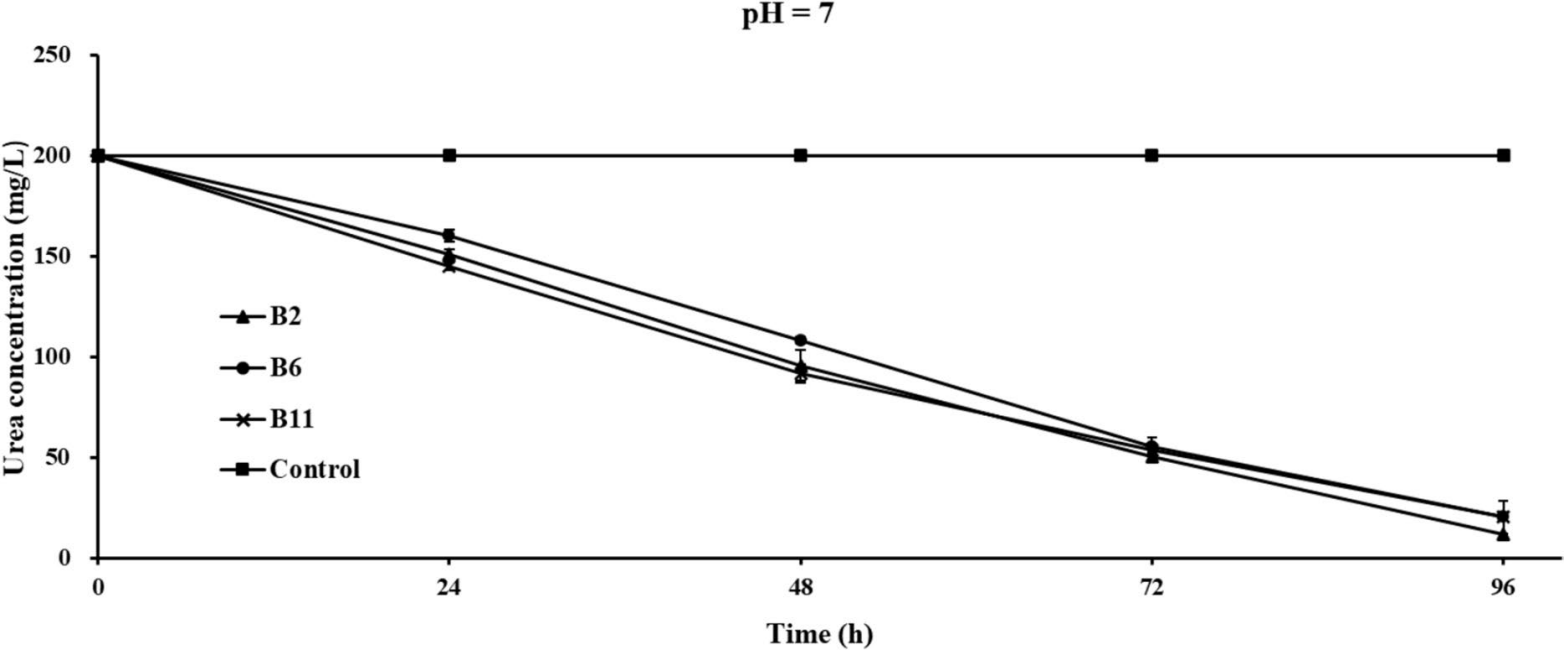
Changes of urea concentrations in the NBU medium of the three selected strains and the control group with time under initial pH 7. The within-group variations for each group (triplicate) of data measured at all periods were significant at the statistical level of p < 0.05 at a 95% confidence interval, except for the control group. Error bars represent standard deviation

**Fig. 3:**
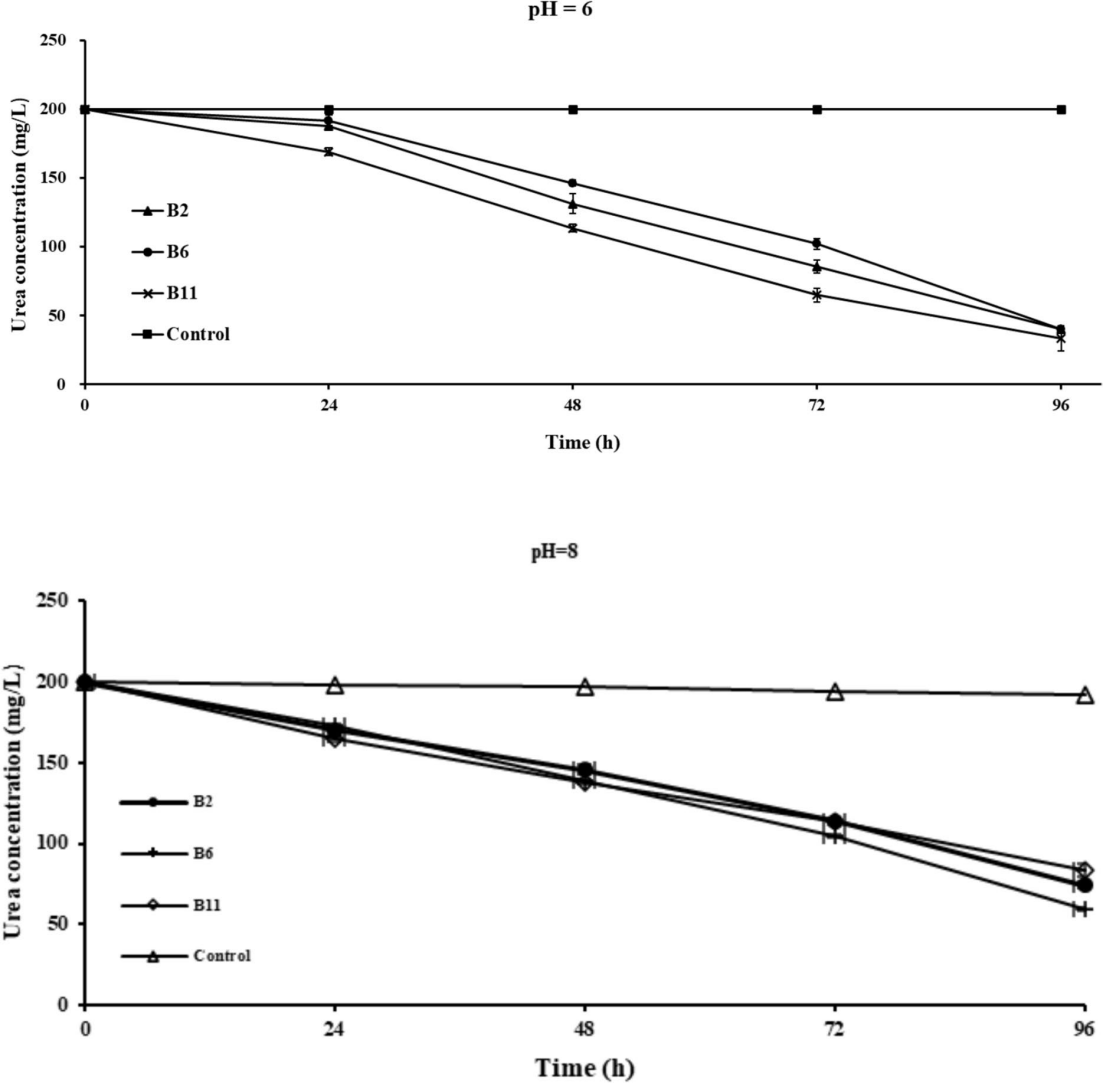
Changes of urea concentrations in the NBU medium of the three selected strains and the control group with time under initial pH of 6 and 8. The within-group variations for each group (triplicate) of data measured at all periods were significant at the statistical level of p < 0.05 at a 95% confidence interval, except for the control group. Error bars represent standard deviation

**Fig. 4:**
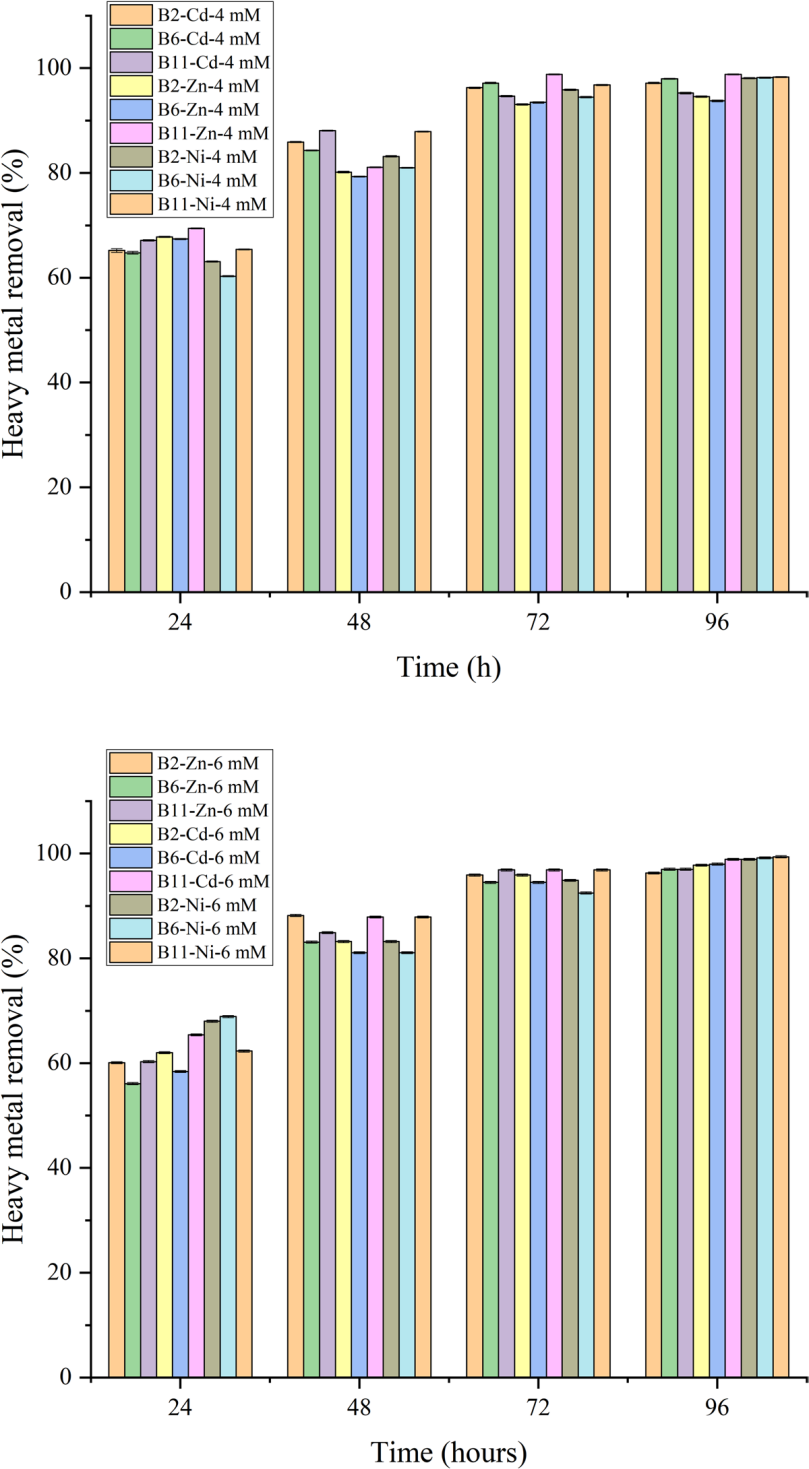
Removal rates of heavy metals by the three selected strains (B2, B6, and B11) at different concentrations (4 mM and 6 mM) of Cd^2+^, Zn^2+^, and Ni^2+^ every 24 h. Error bars indicate standard deviation

The degradation of urea by the three selected ureolytic strains at the corresponding time points of the NBU culture urea concentration under different initial pH is shown in Figs. 2, 3 and 4, and the mean ± standard deviation of the urea concentration in each group represents the error bars. All three selected strains demonstrated strong urea degradation ability over 96 h, regarding net urea degradation, pH 7 > 8 > 6. When the initial pH was 7, the overall urea decomposition trend was consistent for B2 and B11, with urea degrading steadily over 96 h (Fig. 2). However, for B6, the degradation rate slowed down from 48 to 72 h, but from 72 to 96 h, the urea degradation showed a degree of increase.

Most previous studies carried out pH, OD_600_ (bacterial growth), and urease activity tests in conjunction with heavy metal precipitation tests to understand the MICP process. Generally, urea degradation capacity is closely linked to bacterial growth and urease activity (Anbu et al. 2016; Song et al. 2022b). The enzyme activity, i.e., the catalytic rate of the substrate, depends on the enzyme concentration, the substrate concentration, and the enzyme’s affinity to the substrate, which the ambient temperature and pH may influence (Castro-Alonso et al. 2019; Omoregie et al. 2021). In the lag phase of bacterial growth, bacteria maintain a lower level of proliferation and a lower concentration of urease enzyme. Therefore, some previous studies have found that enzyme activity, pH, and OD_600_ are maintained at low levels during the initial stages of the MICP process (Jiang et al. 2019; Zhang et al. 2023b). Interestingly, the urea concentration in our study decreased considerably at 24 h at the pH of 7, implying that the selected strains responded and adapted to the temperature and their physicochemical environment efficiently. For pH 6 and 8, we found that bacteria required an "adaptation phase" before exponentially removing urea from the solution (Fig. 3).

Previous studies have also investigated the growth of ureolytic strains with urea degradation and found that the degradation of urea by ureolytic bacteria in a 20 g/L urea-contained NBU medium at an initial pH of 7 only began 24 h after inoculation (Zhang et al. 2023b). This is consistent with the results obtained at initial pH values of 6 and 8 in the present study. A review conducted by (Krajewska 2018) indicated that urea is decomposed into releasing ammonium ions, which can be measured by quantifying the pH changes, as ammonium ions help increase the solution’s pH. Among the three selected strains, the B2 culture has the lowest urea concentration within 0 to 72 h (50.3 mg/L) at the initial pH of 7, followed by B11 (53.7 mg/L) and B6 (55.4 mg/L). Also, it appears that B2 can adapt faster and start degrading urea in the other two initial pHs, which implies that the capacity of the selected strains for degrading urea during growth ranks in B2 > B11 > B6.

### Removal of heavy metals at different concentrations

**Fig. 5:**
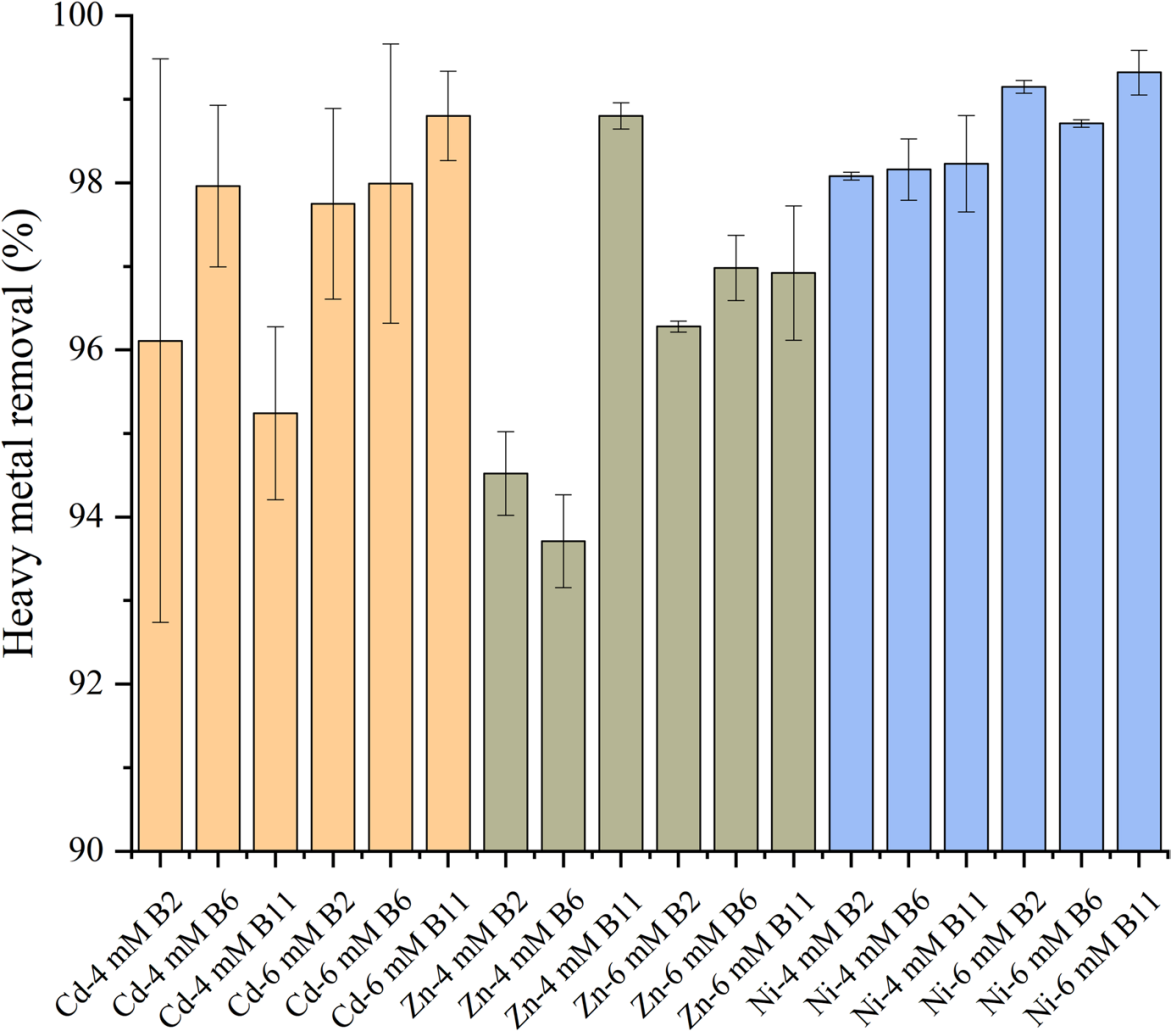
Removal rates of heavy metals by the three selected strains (B2, B6, and B11) at different concentrations (4 mM and 6 mM) of Cd^2+^ (**a**), Zn^2+^ (**b**), and Ni^2+^ (**C**). Error bars indicate standard deviation

The removal rates of heavy metals by B2 (*Bacillus subtilis* HMZC1), B6 (*Bacillus sp.* HMZCSW), and B11 (*Comamonas sp.* HMZC), and their removal capacity are shown in Figs. 4, 5, Table S3, and Table S4. The error bars indicate the mean ± standard deviation of the removal rates of each group of heavy metals (Fig. 4). All three strains showed excellent removal of high concentrations of Cd^2+^, Zn^2+^, and Ni^2+^ in the 96-h heavy metal precipitation experiments. Contrary to the urea degradation experiment, we found that B11 was the most efficient bacteria in removing heavy metals. The highest removal rate occurs in the 6 mM cadmium solution, where B11 removes 98.8% of the cadmium, and B6 removes up to 97.96% of the cadmium in the 4 mM solution. However, for Ni^2+^ and Zn^2+^, higher concentrations of heavy metal solutions are accompanied by higher removal rates, with B11 reaching 96.92% for 6 mM Zn^2+^ and 99.32% removal in 6 mM Ni^2+^ solutions, respectively. Zhang et al. (2023b) discovered that although higher concentrations of heavy metal solutions in the MICP process result in higher amounts of precipitates, this results in lower removal rates, which is inconsistent with our experiment results. We suggest that the influences of heavy metal concentrations on removal rates depend on the tolerance of the strain. Urease-producing bacteria break down urea into ammonia and carbon dioxide, increasing the environment’s pH. This rise in pH promotes the formation of carbonate minerals, effectively trapping heavy metals like cadmium, zinc, and nickel within the solid precipitates (Ji et al. 2024). Additionally, the ammonia produced further enhances the alkaline conditions, making it easier for metals to bind to bacterial surfaces. As seen in Fig. 4, within 24 h, 65% of most heavy metals, on average, were removed by all three bacteria. We found that B6 could only remove around 56% of Zn^2+^- 6 mM and Cd^2+^- 6 mM within 24 h, suggesting an adaptation phase to grow at high concentrations. By 72 h, all the bacteria removed an average of > 93% of all heavy metals, irrespective of their concentrations. To further validate these observations, one-way ANOVA was conducted to compare the performance of the bacterial strains at different time points and heavy metal concentrations. For cadmium, significant differences were observed among the strains at later time points (p < 0.05 for 48 h, 72 h, and 96 h) (Table S3). Similarly, significant differences emerged at 24 h for zinc and nickel and persisted until 96 h (p < 0.05). These results confirm that the strains exhibit distinct patterns of heavy metal removal over time, with B11 showing superior performance at higher concentrations.

A possible explanation lies in B11’s metabolic flexibility. Unlike B2 and B6, which may rely more heavily on urease activity for heavy metal removal, B11 might employ alternative pathways and urease enzymes that enable it to tolerate and remove heavy metals even under highly toxic conditions (Hussein et al. 2024). These mechanisms likely contribute to B11’s superior performance in our heavy metal removal experiments compared to urea degradation experiments. This suggests that heavy metal removal depends more on strain-specific detoxification strategies than urease-mediated pH increases alone.

This suggests that urease-producing bacteria not only adapt to the presence of high metal concentrations but also continuously alter the local environment through sustained urease activity. Urease activity breaks down urea, the resulting increase in carbonate ions provides more opportunities for heavy metals to form stable carbonate minerals. Over time, this process leads to near-complete removal of metals from the solution. Furthermore, the EPS produced by these bacteria likely works with urease activity, acting as a scaffold for mineral nucleation and growth while trapping dissolved metals in their sticky matrix (Zeng et al. 2022). These findings are consistent with previous studies, which observed a similar reduction in heavy metals over 72 h (Sheng et al. 2022; Yina et al. 2021; Khadim et al. 2019; Dong et al. 2019).

Statistical analysis using one-way ANOVA revealed significant differences (p < 0.05) in removal efficiencies among the strains under specific treatment conditions (Table S4). For instance, B11 achieved the highest average removal rate (97.8%), followed by B6 (97.2%) and B2 (96.9%) (Fig. 5).

These differences, though small, highlight the importance of selecting optimal bacterial strains for maximizing heavy metal removal, even when overall performance is consistently high. For example, significant differences were observed for Cd^2+^-4 mM (p = 0.021) and Zn^2+^-4 mM (p = 0.001), where B6 and B11 outperformed B2, respectively. Conversely, no significant differences were found for Ni^2+^-4 mM and Ni^2+^-6 mM (p = 0.654 and p = 0.89), indicating comparable performance across strains for nickel removal. The consistent performance for nickel removal might be linked to its smaller atomic size, allowing it to bind more easily to bacterial surfaces or EPS than larger metals like cadmium. Additionally, urease activity may play a significant role in nickel removal, as the alkaline conditions created by urea hydrolysis enhance nickel’s affinity for carbonate ions, promoting rapid precipitation (Jalilvand et al., 2019). These findings align with prior research emphasizing the role of strain-specific characteristics, such as EPS production and heavy metal affinity, in determining removal efficiencies (Qiao et al. 2021; Bai et al. 2021a).

The effect of heavy metal concentrations used in our study on urease activity and bacterial growth may not be quite as strong; conversely, higher concentrations of heavy metals may be favorable for bacterial surface or EPS capture of heavy metals, i.e., higher opportunities to compete with binding sites (Zhao et al. 2017; Zeng et al. 2022). The average removal of all heavy metals by the different strains in descending order was B11 (98.22%) > B6 (97.83%) > B2 (97.15%), and the average removal rates of the different heavy metals ranked from highest to lowest in the order of Ni^2+^ (98.68%) > Cd^2+^ (98.14%) > Zn^2+^ (96.37%). All three strains demonstrated exceptionally high removal rates, exceeding 93% for all tested heavy metals. For instance, B11 achieved the highest average removal rate of 98.22%, followed by B6 (97.83%) and B2 (97.15%). These findings highlight the importance of selecting optimal bacterial strains for maximizing heavy metal removal, even when overall performance is consistently high. Previously, Jalilvand et al. (2019) investigated the MICP process for Pb^2+^, Cd^2+^, and Zn^2+^ removal and found that the average removal rates for Zn^2+^ (77.51%) and Cd^2+^ (80.63%) demonstrated higher efficiency for Cd^2+^ despite its more significant toxicity compared to Zn^2+^. This could be attributed to the difference between the atomic structures. Disi et al. (2022) considered that the larger the atomic radius of the heavy metal, the stronger the ability of the heavy metal to compete with the binding site. These results underscore the potential of urease-producing bacteria, particularly B11, for bioremediation of heavy metal-contaminated environments. Scaling up this process could offer a sustainable wastewater treatment and ecological restoration solution.

### Characterization analysis

**Fig. 6:**
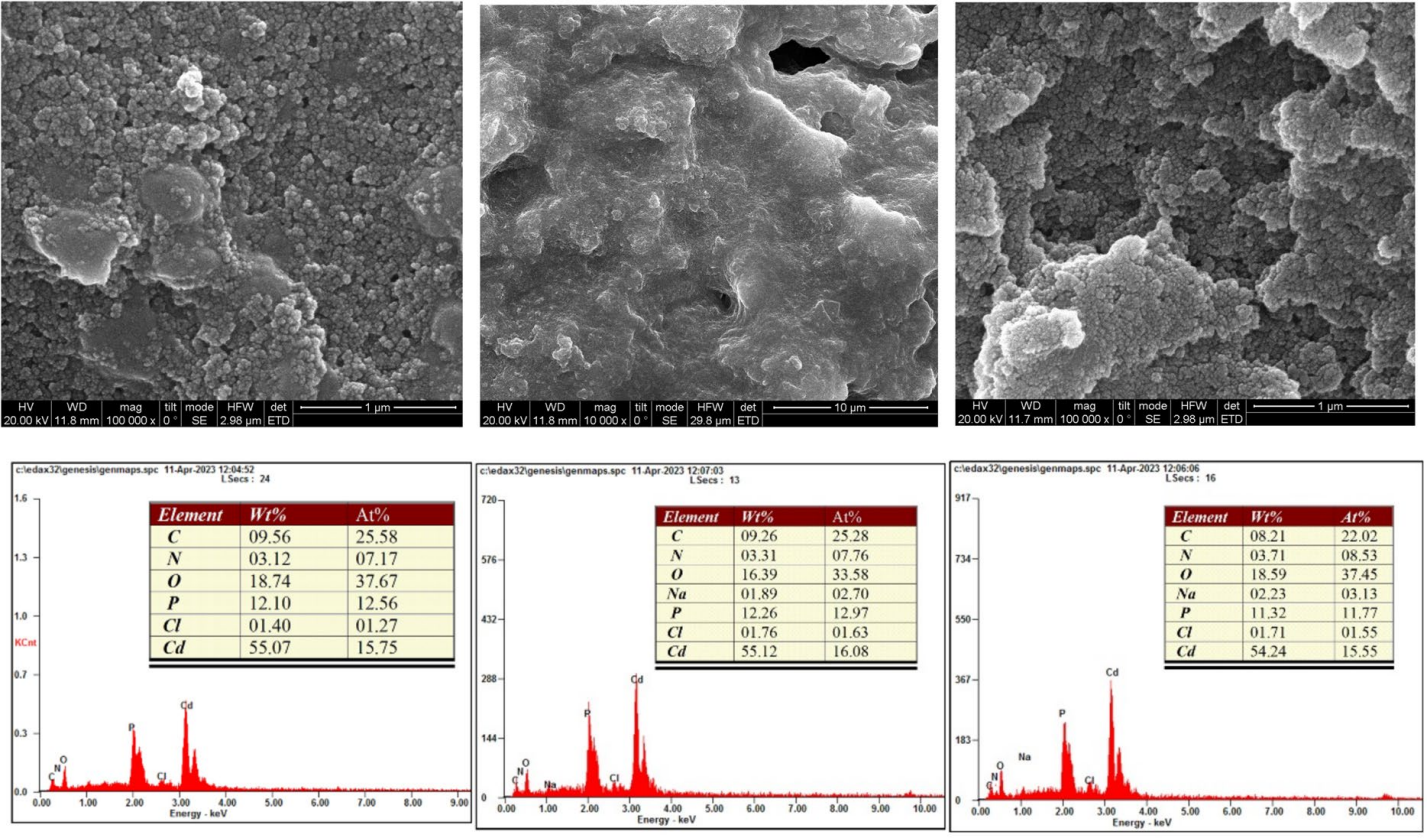
SEM images (Top panel) of the minerals formed by B2, B6, and B11 in the presence of Cd. EDS (bottom panel) illustrates the elemental composition of the formed minerals. The tables indicate the wt% and atom % of each element

**Fig. 7:**
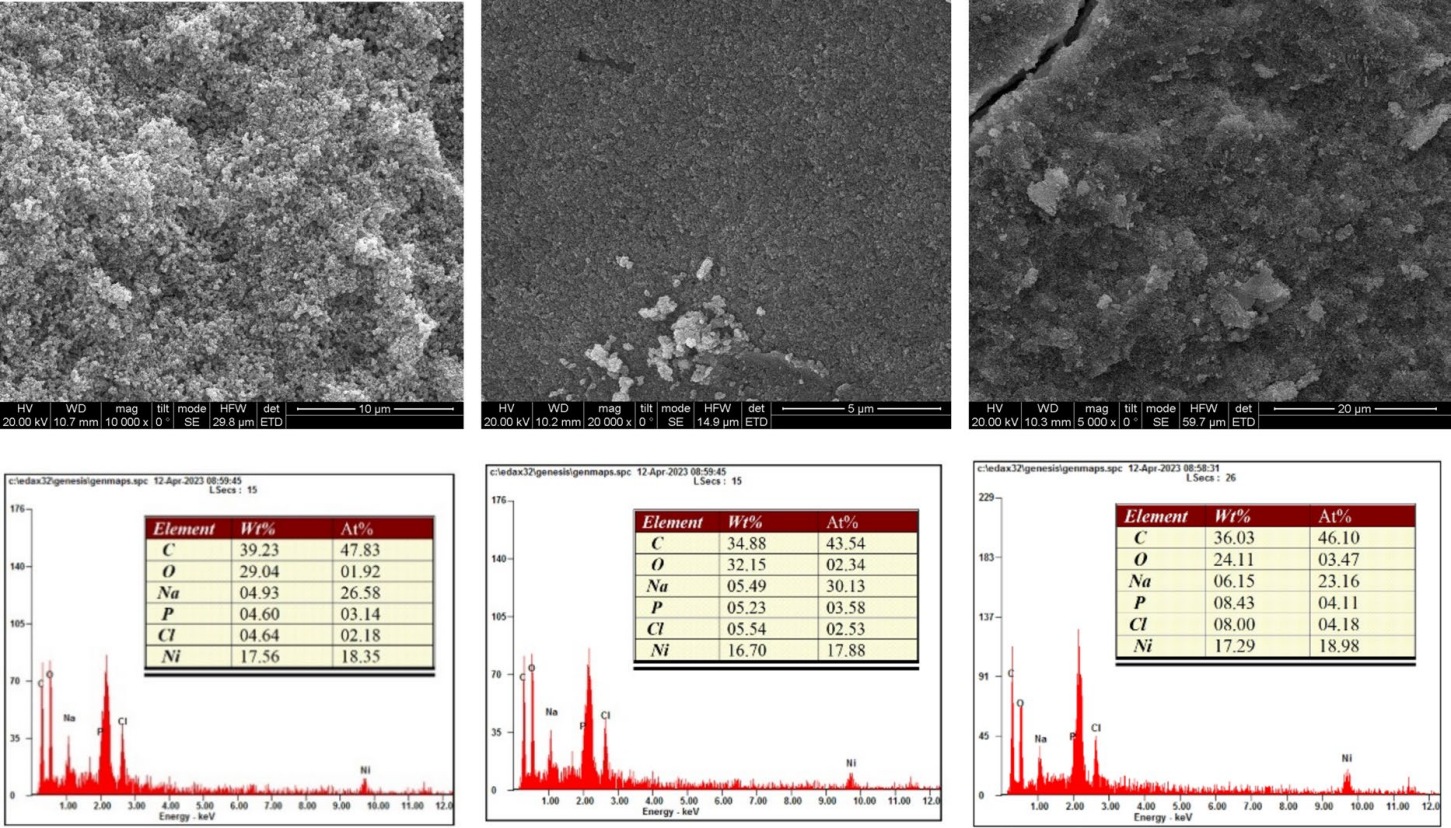
SEM images (Top panel) of the minerals formed by B2, B6, and B11 in the presence of Ni. EDS (bottom panel) illustrates the elemental composition of the formed minerals. The tables indicate the wt% and atom % of each element

**Fig. 8:**
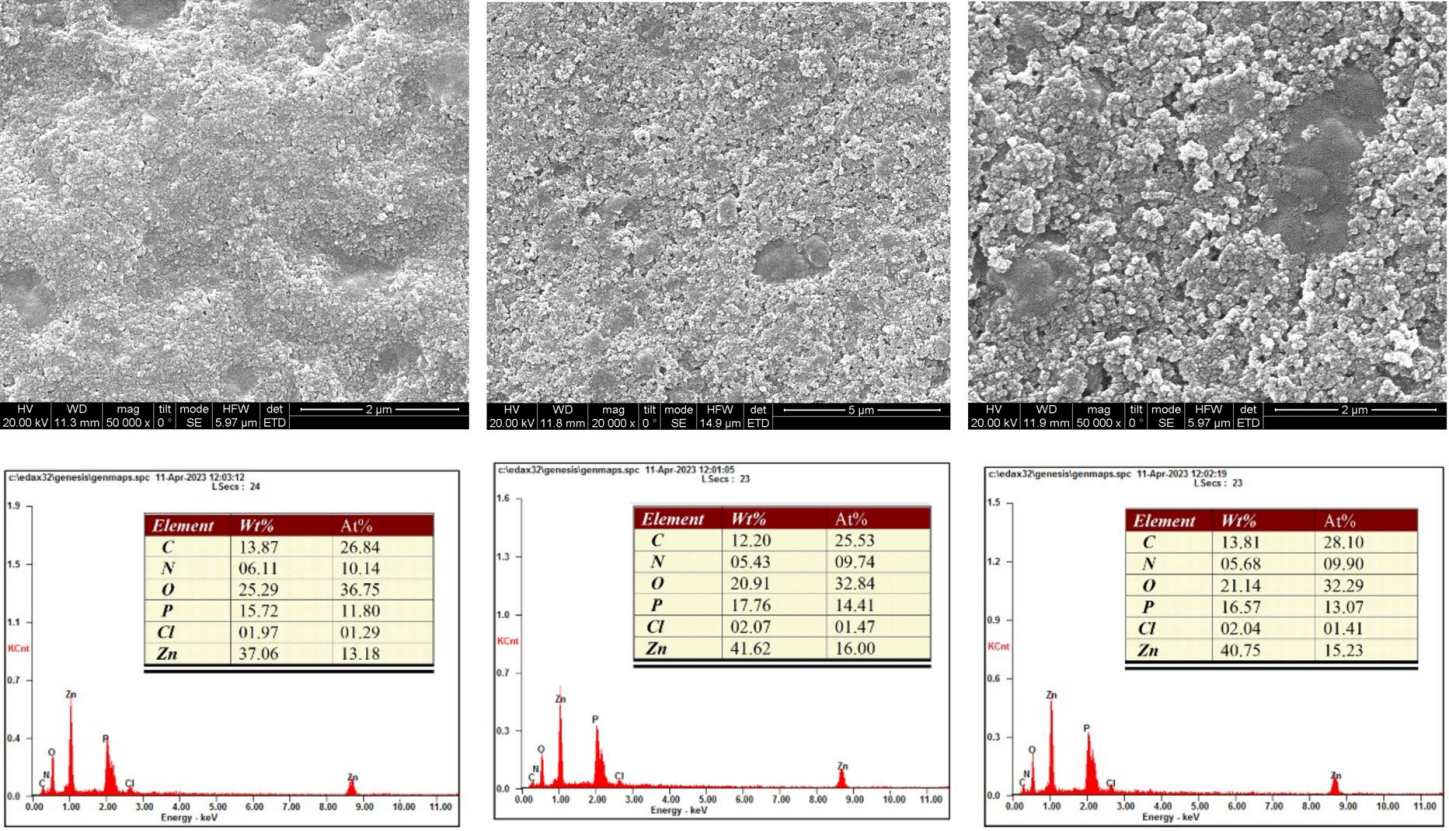
SEM images (Top panel) of the minerals formed by B2, B6, and B11 in the presence of Zn. EDS (bottom panel) illustrates the elemental composition of the formed minerals. The tables indicate the wt% and atom % of each element

The SEM analysis of the bacterial precipitation of cadmium, zinc, and nickel carbonate revealed intriguing morphological changes and elemental composition shifts within the biomineralized material. Figures 6, 7 and 8 show the formation of porous and irregular aggregates of heavy metal carbonate. This porous structure indicates microbial involvement in carbonate precipitation, which can be a nucleation site for forming carbonate minerals. These porous structures are formed because bacteria create local environments that favor mineral growth by secreting the urease enzyme that precipitates carbonate and binds with metal ions (Cd^2+^, Ni^2+^, and Zn^2+^). This process not only traps metals but also makes the precipitates more stable. The trapped metals are less likely to dissolve into the environment, reducing their potential toxicity (Taharia et al. 2024). Additionally, the irregular and porous nature of the aggregates suggests that the bacteria may continue to influence mineral growth over time, leading to ongoing changes in structure and composition. Several researchers also observed these heavy metal carbonates (Sheng et al. 2022; Khadim et al. 2019; Dong et al. 2019; Ji et al. 2024). EDS analysis (Figs. 6, 7 and 8) showed a substantial increase in Cd and Zn concentration in the biomineralized material, confirming the incorporation of cadmium and zinc ions into the precipitate (Zhang et al. 2023c; Disi et al. 2022; Qiao et al. 2021). This suggests that the bacteria effectively captured these metals, potentially by adsorbing them to their surfaces and trapping them. We found that Ni^2+^ had a lower percentage than the other heavy metals. Researchers also previously observed this for Nickel carbonate (Khadim et al. 2019). Despite its efficient removal, the lower percentage of Ni^2+^ in the biomineralized material highlights the dominance of surface interactions over carbonate precipitation kinetics in the MICP process (Liu et al. 2021). The carbon (C) signal suggests that bacterial cells were actively involved in precipitation, potentially acting as nucleation sites for carbonate mineral growth (Bai et al. 2021a).

**Fig. 9:**
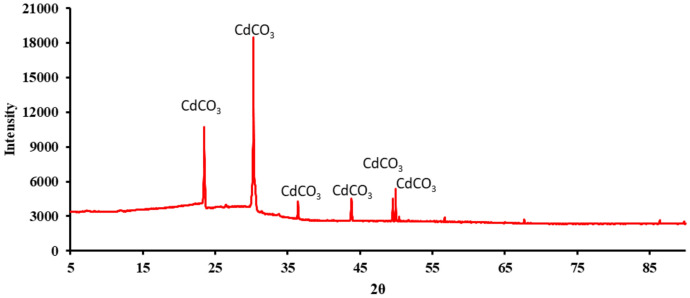
XRD for cadmium carbonate precipitated by the bacteria

**Fig. 10:**
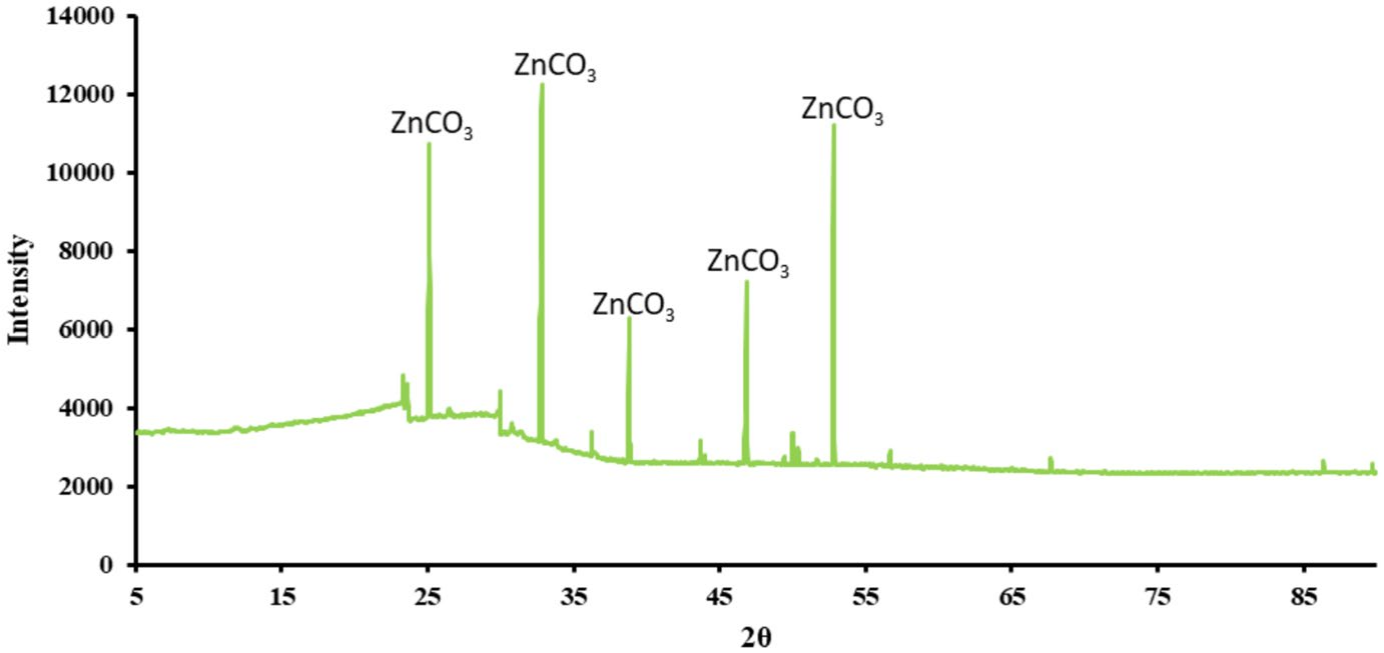
XRD for zinc carbonate precipitated by the bacteria

**Fig. 11:**
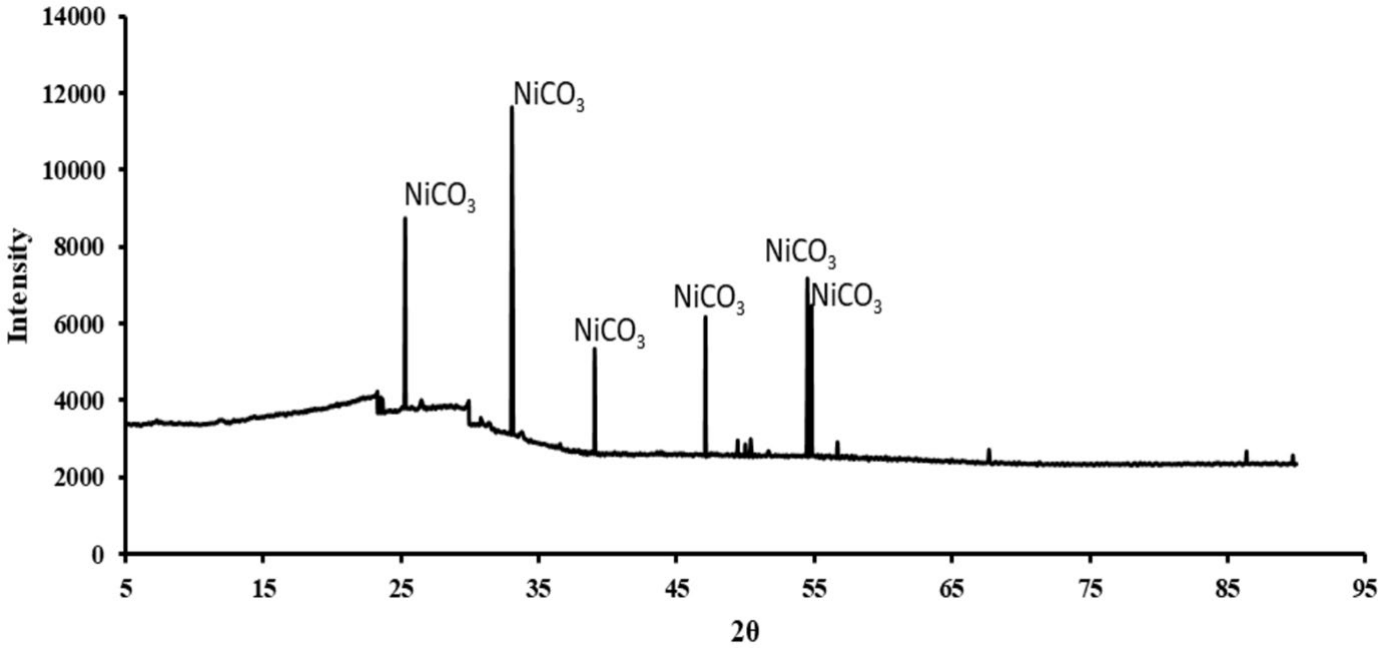
XRD for Nickel carbonate precipitated by the bacteria

The XRD pattern for the biomineralized Cadmium Carbonate (Fig. 9), Zinc Carbonate (Fig. 10), and Nickel carbonate (Fig. 11) precipitated by the bacteria was compared with IDCC 00–042-1342, 00–001-1036, and 00–012-0771 (Kabekkodu et al. 2024). Similar diffraction peaks were identified at 2θ angles, representing the precipitation of these heavy metal carbonates. We compared our peaks with existing literature for further validation and found them to be similar (Zeng et al. 2022; Yina et al. 2021; Khadim et al. 2019; Baddar et al. 2021, Jalilvand et al., 2019). Sharp and well-defined peaks consistent with the known crystalline structure for these heavy metal carbonates were obtained. Previous studies have also reported the precipitation of heavy metal carbonates, validating the findings that carbonate precipitated by bacteria can bind with divalent heavy metal ions to form heavy metal carbonate crystals. (Kim et al. 2021; Zhang et al. 2022, 2024; Zeng et al. 2022). The crystallinity index was not explicitly calculated in this study. However, the XRD peaks obtained from the precipitates were compared with international standard peak data for heavy metal carbonates; this approach has been commonly applied in MICP heavy metal studies (Ji et al. 2024; Zhang et al. 2024). This comparison confirmed the presence of crystalline phases corresponding to heavy metal carbonates, such as zinc carbonate, cadmium carbonate, and nickel carbonate. While the crystallinity index was not determined, the clear identification of these phases supports the effectiveness of the MICP process in forming stable carbonate precipitates. Future studies will include a detailed analysis of the crystallinity index to evaluate the structural properties of the precipitates further.

## Conclusion

The increasing concentration of heavy metal pollution in ecosystems has provoked a search for innovative, effective, and eco-friendly remediation strategies. The present study illuminated the potential application of Microbial Induced Calcium Carbonate Precipitation (MICP) in addressing this environmental dilemma. By isolating and testing ureolytic bacterial strains B2 (*Bacillus subtilis* HMZC1), B6 (*Bacillus sp.* HMZCSW), and B11 (*Comamonas sp.* HMZC) for their respective efficiencies in the biomineralization of calcium carbonate and simultaneous mitigation of heavy metal ions (Cd, Zn, and Ni) from polluted water, this research establishes a foundation for the potential application of MICP in bioremediation.

B2 was the most efficient ureolytic bacteria, followed by B11 and B6 in urea degradation at pH 6, 7, and 8. We found pH 7 to be the optimum pH for urea degradation. However, B11 emerged as a potent candidate among the bacterial strains studied, demonstrating a substantial removal efficiency reaching up to > 95% for cadmium ions under optimal conditions. Our study demonstrates that urease-producing bacteria B11, B6, and B2 achieve > 93% removal of Cd, Zn, and Ni within 72 h. B11 excels with 98.8% Cd removal at 6 mM and > 99% Ni removal. Statistical analysis (p < 0.05) confirms significant strain-specific differences, highlighting B11’s superiority for bioremediation applications. This establishes a distinct variance in the heavy metal mitigation capabilities across different bacterial strains, suggesting preferential applicability for different strains in context-specific bioremediation scenarios.

SEM analyses revealed that B11 facilitated the formation of metal precipitates, thereby providing a morphological basis for understanding the immobilization of heavy metals within the biomineralized structures. Furthermore, XRD and EDS analyses corroborated that the incorporation of metals into the carbonate matrix was stable and reliable, implicating the precipitates’ long-term stability and suitability for durable, sustainable heavy metal removal. This study highlights the potential of urease-producing bacteria, particularly *Comamonas sp.* HMZC, for bioremediation of heavy metal-contaminated environments.

## Future directions

Future research should optimize the process for large-scale applications, including field trials, to assess its feasibility under real-world conditions. Key areas of investigation will include:

*Scaling Up*: Conducting pilot-scale experiments to evaluate the scalability of MICP for industrial or environmental applications.

*Cost-Effectiveness*: Analyzing the economic feasibility of implementing MICP compared to conventional remediation techniques.

*Long-Term Stability*: Assessing the durability of metal carbonates over extended periods to ensure sustained immobilization of heavy metals.

*Challenges*: Addressing potential limitations, such as variability in bacterial activity, sensitivity to environmental conditions, and the need for standardized protocols.

Although preliminary, this research promotes the potential of harnessing microbiological processes with biomineralization to develop environmentally efficient, sustainable strategies for addressing the pervasive issue of heavy metal pollution, thereby contributing to a cleaner, safer, and more sustainable future.

## Supplementary Information

Below is the link to the electronic supplementary material.

Supplementary file1 (DOCX 166 KB)

## Data Availability

Data will be made available at a reasonable request to the corresponding author.
