# Supplementary material for: Removal of high concentrations of zinc, cadmium, and nickel heavy metals by Bacillus and Comamonas through microbially induced carbonate precipitation
Source: Biodegradation. 2025 May 5;36(3):40. doi: 10.1007/s10532-025-10131-7 (PMC12053368; doi:10.1007/s10532-025-10131-7)
Supplement: Supplementary file 1 — Supplementary file1 (DOCX 166 KB) [file 10532_2025_10131_MOESM1_ESM.docx]

**
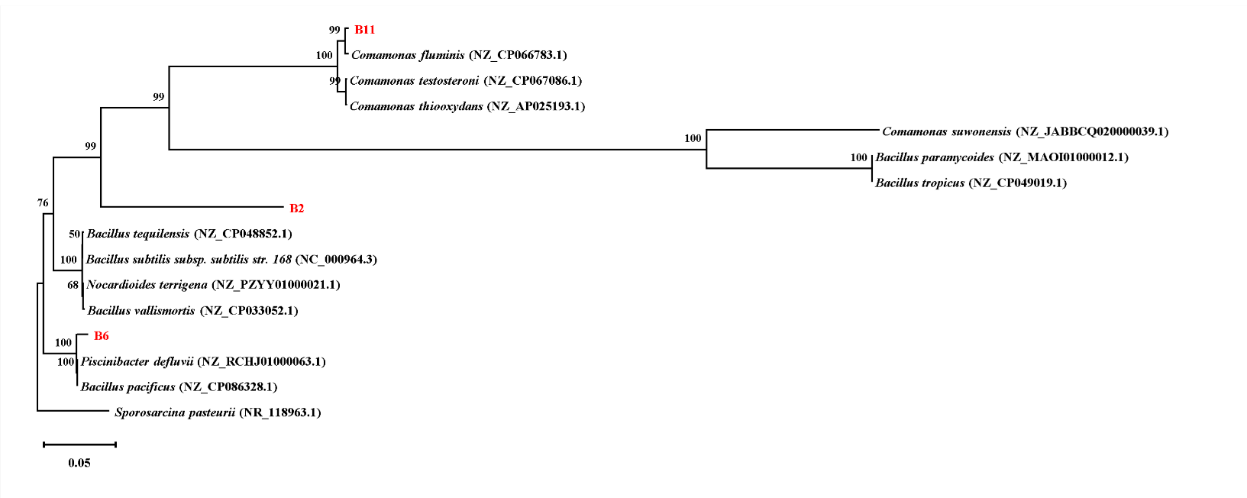
**

**Figure S1.** The sequences of the three isolates based on 16s rRNA sequencing results were aligned with known bacterial strains, and a phylogenetic tree was constructed using the neighbor-joining method, with sequence markers indicated by "Taxonomic Name (Sequence ID)" and the scale bar indicating genetic distance.

**Table S1.** The types of heavy metals and focused bacteria strains in previous studies of heavy metal removal by ureolytic MICP processes. NBU= Nutrient Broth-Urea.

| Heavy metals in the NBU solution | Name of the bacteria | References |
| --- | --- | --- |
| CuSO_4_·5H_2_O | *Kocuria* *flavus* | (Achal et al., 2011) |
| CdCl_2_·5H_2_O, PbCl_2_, CuCl_2_ | *Viridibacillus arenosi B-21, Sporosarcina soli B-22, Enterobacter cloacae KJ-46, and E. cloacae KJ-47* | (Kang et al., 2016) |
| K_2_Cr_2_O_7,_ PbCl_2_ | *Staphylococcus epidermidis HJ2* | (He et al., 2019) |
| ZnCl_2_, Pb (NO_3_)_2_, CdCl_2_ | *Variovorax boronicumulans*, *Stenotrophomonas rhizophila,* and *Sporosarcina pasteurii* | (Jalilvand et al., 2019a) |
| Pb (NO_3_)_2_ | *Sporosarcina pasteurii* | (Jiang et al., 2019) |
| CdCl_2_.5H_2_O | *Sporosarcina pasteurii* | (Ghorbanzadeh et al., 2020) |
| CuCl_2_⋅2H_2_O, ZnCl_2_, Pb (CH_3_CO_2_) _2_⋅3H_2_O, Cd (C_2_H_3_O_2_)_2_⋅2H_2_O, SrCl_2_⋅6H_2_O | *Sporosarcina pasteurii* | (Kim et al., 2021a) |
| CdCl_2_, ZnCl_2_, NiCl_2_, CuCl_2_ | *Lysinibacillus* sp., *Pseudochrobactrum* sp., and *Sporosarcina* sp. | (Qiao et al., 2021) |
| PbCl_2_ | *Exiguobacterium* sp. | (Bai et al., 2021a) |
| CaCl_2_, CdCl_2_ | *Sporosarcina ureilytica*ML-2 | (Zeng et al., 2022) |
| CaCl_2_·2H_2_O, CdCl_2_ | *Serratia* sp., *Acinetobacter* sp. | (Diez-Marulanda and Brandão, 2023) |

**Table S2.** The concentrations of heavy metals were studied, the duration of heavy metal precipitation experiments was determined, and removal rates were obtained in previous ureolytic MICP of heavy metals research.

| Concentration of the HMs | Duration | Removability | References |
| --- | --- | --- | --- |
| 100, 250, 500, 750 and 1000 mg/L | 120 h | 92% (250 mg/L), 95% (500 mg/L), 96% (750 mg/L), 97% (1000 mg/L) | (Achal et al., 2011) |
| 2 mM | 48 h | 98.25% (Pb), 85.39% (Cd), and 5.56% (Cu) | (Kang et al., 2016) |
| 25 mg/L | 168 h | 76.8% (Cr), 86% (Pb) | (He et al., 2019) |
| 2 mM | 72 h | Average: Zn (77.51%), Cd (80.63%), Pb (96.96%) | (Jalilvand et al., 2019a) |
| 0-50 mM | 48 h | Close to 100% | (Jiang et al., 2019) |
| 56.2, 112.4, and 224.8 mg/L | 48 h | 0.218, 0.225 and 0.486 mg/L | (Ghorbanzadeh et al., 2020) |
| 10, 20, 40, 80, 160 mg/L | 12 h | kp-4: 95.17% (Cd), 95.26% (Cu), 85.92% (Zn), 42.81% (Ni)  kp-22: 99.99% (Cd), 77.54% (Cu), 84.76% (Zn), 53.63% (Ni) | (Qiao et al., 2021) |
| 0.01, 0.05, 0.1, 0.5, and 1 mM (Cu, Zn, Pb, and Cd)  1, 5, 10, 20 and 30 mM (Sr) | 336 h | 60% (Cu), 30% (Zn), 94.5% (Pb), 43.2% (Cd), Close to 100% (Sr) | (Kim et al., 2021a) |
| 1 mM | 120 h | 98.1% (3% salinity) | (Bai et al., 2021a) |
| Pb: 0-1000 ppm  Cu: 0-100 ppm | 48 h | 98.75% (200 ppm Pb)  92.3% (20 ppm Cu) | (Li et al., 2021) |
| 1 mM | 48 h | Close to 100% | (Zeng et al., 2022) |
| 0.05 mM | 144 h | *Serratia* sp.: 99.70% and 99.62%  *Acinetobacter* sp.: 91.23% | (Diez-Marulanda and Brandão, 2023) |

**Table S3**. Summary of One-Way ANOVA Results for Heavy Metal Removal by Bacterial Strains (B2, B6, B11) at Different Concentrations (4 mM and 6 mM) and Time Points (0h, 24h, 48h, 72h, 96h). The table shows the p-value in heavy metal removal efficiency among the strains for cadmium (Cd), zinc (Zn), and nickel (Ni). Significant differences are indicated by p < 0.05

| **Heavy Metal** | **Concentration (mM)** | **Time (h)** | **p-value** |
| --- | --- | --- | --- |
| Cd | 4 | 0 | p > 0.05 |
| Cd | 4 | 24 | p < 0.05 |
| Cd | 4 | 48 | p < 0.05 |
| Cd | 4 | 72 | p < 0.05 |
| Cd | 4 | 96 | p < 0.05 |
| Cd | 6 | 0 | p > 0.05 |
| Cd | 6 | 24 | p < 0.05 |
| Cd | 6 | 48 | p < 0.05 |
| Cd | 6 | 72 | p < 0.05 |
| Cd | 6 | 96 | p < 0.05 |
| Zn | 4 | 0 | p > 0.05 |
| Zn | 4 | 24 | p < 0.05 |
| Zn | 4 | 48 | p < 0.05 |
| Zn | 4 | 72 | p < 0.05 |
| Zn | 4 | 96 | p < 0.05 |
| Zn | 6 | 0 | p > 0.05 |
| Zn | 6 | 24 | p < 0.05 |
| Zn | 6 | 48 | p < 0.05 |
| Zn | 6 | 72 | p < 0.05 |
| Zn | 6 | 96 | p < 0.05 |
| Ni | 4 | 0 | p > 0.05 |
| Ni | 4 | 24 | p < 0.05 |
| Ni | 4 | 48 | p < 0.05 |
| Ni | 4 | 72 | p < 0.05 |
| Ni | 4 | 96 | p < 0.05 |
| Ni | 6 | 0 | p > 0.05 |
| Ni | 6 | 24 | p < 0.05 |
| Ni | 6 | 48 | p < 0.05 |
| Ni | 6 | 72 | p < 0.05 |
| Ni | 6 | 96 | p < 0.05 |

**Table S4.** The mean values and standard deviations (SD) for bacteria B2, B6, and B11 under various treatment conditions. One-way analysis of variance (ANOVA) was conducted to assess statistically significant differences between the groups, with p-values indicating significance at the 0.05 level.

| Group | B2- Mean (SD) | B6- Mean (SD) | B11- Mean (SD) | p-value |
| --- | --- | --- | --- | --- |
| Cd-4 mM | 96.11 (3.372) | 97.96 (0.968) | 95.24 (1.035) | 0.021 |
| Cd-6 mM | 97.75 (1.143) | 97.99 (1.669) | 98.8 (0.535) | 0.324 |
| Zn-4 mM | 94.52 (0.499) | 93.71 (0.553) | 98.8 (0.158) | 0.001 |
| Zn-6 mM | 96.28 (0.068) | 96.98 (0.390) | 96.92 (0.802) | 0.456 |
| Ni-4 mM | 98.08 (0.048) | 98.16 (0.368) | 98.23 (0.576) | 0.654 |
| Ni-6 mM | 99.15 (0.076) | 98.71 (0.045) | 99.32 (0.267) | 0.89 |
